# *Pandanus amaryllifolius* and *Tectona grandis* Extracts Improve Fetal Outcomes in Streptozotocin-Induced Gestational Diabetes in Rats

**DOI:** 10.3390/ijms27020857

**Published:** 2026-01-15

**Authors:** Sasitorn Kerdsuknirund, Pakanit Kupittayanant, Pattama Tongdee, Porntip Nimkuntod, Sajeera Kupittayanant

**Affiliations:** 1 School of Preclinical Sciences, Institute of Science, Suranaree University of Technology, Nakhon Ratchasima 30000, Thailand; sasi.kerdsuknirund@gmail.com; 2 School of Animal Technology and Innovation, Institute of Agricultural Technology, Suranaree University of Technology, Nakhon Ratchasima 30000, Thailand; pakanit@sut.ac.th; 3 School of Obstetrics and Gynecology, Institute of Medicine, Suranaree University of Technology, Nakhon Ratchasima 30000, Thailand; pattama_t@sut.ac.th; 4 Suranaree University of Technology Hospital, Nakhon Ratchasima 30000, Thailand; porntipnimk@sut.ac.th; 5 School of Internal Medicine, Institute of Medicine, Suranaree University of Technology, Nakhon Ratchasima 30000, Thailand

**Keywords:** gestational diabetes mellitus, *Pandanus amaryllifolius*, *Tectona grandis*, polyherbal formulation, placenta

## Abstract

Gestational diabetes mellitus (GDM) causes adverse effects on both mothers and offspring. This study investigated the effects of a polyherbal formulation combining *Pandanus amaryllifolius* root and *Tectona grandis* leaf extracts on maternal and fetal outcomes in streptozotocin (STZ)-induced GDM rats, compared with metformin. Pregnant rats were assigned to a non-diabetic reference group and diabetic groups, including an untreated diabetic group (negative control), a metformin-treated group (positive control), and diabetic groups treated with low, medium, or high doses of the pandan–teak formulation from gestation day 7 to 21. Medium and high doses significantly increased maternal body weight and pancreatic mass index (*p* < 0.05) without altering maternal glycemia or insulin levels. Fetal weight increased at medium and high doses, whereas crown–rump length increased only at the high dose. Placental index and fetal glucose levels decreased in a dose-dependent manner (*p* < 0.05), with no significant change in implantation loss. These findings suggest that the pandan–teak formulation may exert complementary actions that support placental–fetal glucose regulation and fetal growth while maintaining maternal glycemic stability, indicating its potential as a plant-based adjunct approach for gestational diabetes focused on fetal protection.

## 1. Introduction

Gestational diabetes mellitus (GDM) is a pregnancy-specific metabolic disorder characterized by impaired glucose regulation due to heightened insulin resistance and insufficient pancreatic β-cell compensation [1]. Unlike type 1 diabetes, GDM results from pregnancy-related hormonal and metabolic alterations rather than autoimmune β-cell destruction. The condition poses serious risks for both mother and fetus, including preeclampsia, cesarean delivery, macrosomia, neonatal hypoglycemia, and long-term metabolic disease in the offspring. As a result, there is growing interest in complementary therapies—particularly medicinal plants with hypoglycemic properties [2]. Women who experience GDM are also at greater risk of developing type 2 diabetes later in life [2].

Conventional treatment emphasizes dietary adjustment, physical activity, and pharmacological therapy—typically insulin or metformin—to normalize maternal glycemia. Although effective, these agents carry important limitations, such as maternal hypoglycemia, gastrointestinal side effects, and uncertain fetal safety profiles [3]. These concerns, combined with rising global interest in phytotherapeutics, have encouraged exploration of safe, plant-based adjuncts that could protect the mother–fetus dyad without disturbing normal gestational physiology.

A variety of medicinal plants have demonstrated hypoglycemic, antioxidant, or insulin-sensitizing activities. Species such as *Trigonella foenum-graecum* (fenugreek), *Momordica charantia* (bitter melon), *Cinnamomum verum* (cinnamon), and *Aloe vera* have been investigated for diabetes management [4]. In Southeast Asia, *Pandanus amaryllifolius* (pandan) and *Tectona grandis* (teak) are among the most widely used plants for glycemic control. Pandan contains diverse phytochemicals—including flavonoids, terpenoids, and alkaloids—that collectively exhibit antihyperglycemic, antioxidant, and insulinotropic properties [5]. Experimental studies report its ability to inhibit α-amylase activity, enhance glucose uptake, increase insulin secretion, and improve hepatic glycogen storage [6,7]. Teak leaf extract is rich in polyphenolic antioxidants and naphthoquinones capable of reducing oxidative stress, inflammation, and β-cell injury [8,9]. Both plants have been used traditionally to alleviate diabetic symptoms, yet their concurrent use during pregnancy has not been systematically evaluated.

Because GDM is multifactorial, combining botanicals with complementary actions could achieve more balanced metabolic effects while lowering the required dose of each individual extract [10]. Pandan primarily supports glycemic regulation, whereas teak provides strong antioxidant and anti-inflammatory protection. Co-administration may therefore produce synergistic benefits targeting both maternal metabolic stability and placental–fetal protection [11].

Among inflammatory mediators implicated in GDM, tumor necrosis factor-α (TNF-α) is a key driver of insulin resistance. Elevated maternal TNF-α correlates positively with fasting glucose and homeostatic model assessment of insulin resistance (HOMA-IR) [12]. By impairing insulin receptor signaling and promoting oxidative stress, TNF-α contributes to both maternal hyperglycemia and placental dysfunction [13]. Preclinical streptozotocin (STZ) models reproduce this pattern, showing heightened TNF-α in maternal serum and placental tissue [14]. Therefore, agents that attenuate oxidative and inflammatory stress—such as teak-derived polyphenols—may indirectly normalize placental and fetal metabolism.

Maternal hyperglycemia exposes the fetus to excessive glucose, provoking compensatory fetal β-cell hyperplasia, hyperinsulinemia, and accelerated adipose growth (macrosomia). In contrast, microangiopathic complications of maternal diabetes or placental insufficiency may restrict nutrient transfer, leading instead to fetal growth restriction. Both extremes share a common pathophysiology of disrupted placental glucose transport, oxidative stress, and mitochondrial dysfunction [15]. Importantly, several plant-derived compounds can cross the placenta and modulate fetal metabolism. For instance, paeoniflorin from *Paeonia lactiflora* lowered maternal and fetal glucose levels in GDM rats [16], while cinnamaldehyde from Cinnamomum species improved maternal–fetal glucose and insulin profiles [17]. These findings suggest that certain phytochemicals can influence fetal glycemic regulation independently of maternal glucose control.

Given this context, polyherbal formulations designed for pregnancy merit investigation not only for maternal benefits but also for their capacity to normalize placental and fetal physiology. The pandan–teak combination represents such a candidate: pandan’s insulinotropic and α-glucosidase-inhibitory activities may stabilize maternal energy supply, whereas teak’s antioxidant constituents could protect the placenta and fetal tissues from oxidative damage. Together, these mechanisms could enhance placental efficiency and restore normal fetal growth even when maternal glycemia remains moderately elevated.

Accordingly, the present study employed a validated STZ-induced gestational diabetes model in rats to evaluate the combined effects of *Pandanus amaryllifolius* root and *Tectona grandis* leaf extracts on maternal and fetal outcomes. We hypothesized that the polyherbal formulation would improve fetal growth parameters and reduce fetal glucose levels without significantly altering maternal blood glucose, indicating a selective placental–fetal modulatory effect. By comparing multiple dose levels with metformin treatment, this work aimed to establish experimental evidence for a safe, plant-based adjunct capable of enhancing fetal outcomes in diabetic pregnancy.

## 2. Results

### 2.1. Phytochemical Analysis of Pandan and Teak Extract Formulation

**Figure 1:**
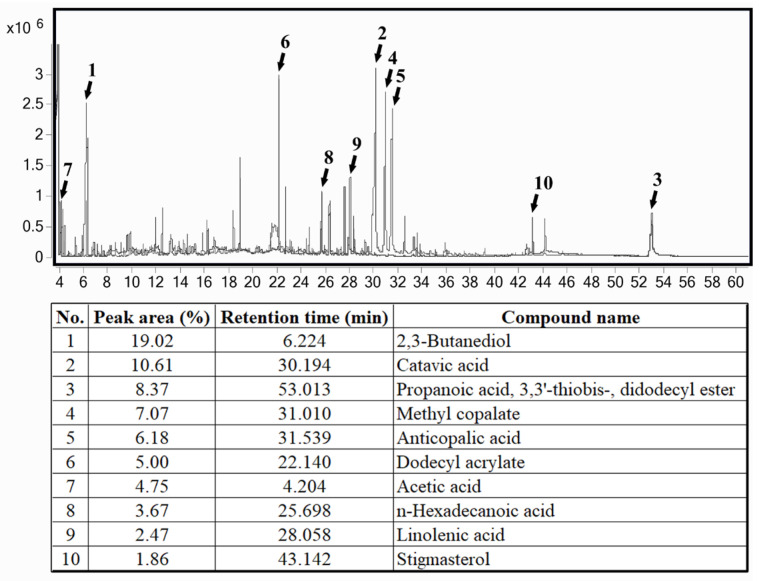
Gas Chromatography–Mass Spectrometry (GC/MS) chromatogram of the combined extract formulation containing *Pandanus amaryllifolius* root and *Tectona grandis* leaf. The x-axis represents the retention time (in minutes), while the y-axis indicates signal intensity. Peaks are numbered and labeled, corresponding to specific compounds detailed in the accompanying table. The most abundant constituents include 2,3-Butanediol (retention time: 6.224 min, peak area: 19.02%) and catavic acid (30.194 min, 10.61%). This overlay chromatogram offers an integrated view of the phytochemical composition of the combined extract, supporting its potential biological activities.

The Gas Chromatography–Mass Spectrometry (GC/MS) analysis of the combined extract formulation derived from *Pandanus amaryllifolius* root and *Tectona grandis* leaf revealed a distinct phytochemical profile, as illustrated in Figure 1. The ten most abundant compounds identified in the formulation included 2,3-Butanediol (19.02%), catavic acid (10.61%), propanoic acid, 3,3′-thiobis-, didodecyl ester (8.37%), methyl copalate (7.07%), anticopalic acid (6.18%), acetic acid (4.75%), dodecyl acrylate (5.00%), *n*-hexadecanoic acid (3.67%), linolenic acid (2.47%), and stigmasterol (1.86%).

The detection of these bioactive constituents suggests that the combined extract may exert significant antioxidant and antidiabetic effects. The overlay of chromatograms in this analysis consolidates the chemical composition of the two botanicals into a single visual representation, providing a clearer understanding of the collective phytochemical profile of the formulation.

Catavic acid and copalic acid derivatives (including methyl copalate and anticopalic acid) are structurally related diterpenoids belonging to the copalyl-type resin acids. Differences in nomenclature reflect variations in structural annotation and reporting across databases and literature sources.

### 2.2. Maternal Fasting Glucose, Insulin, and TNF-α Levels

**Figure 2:**
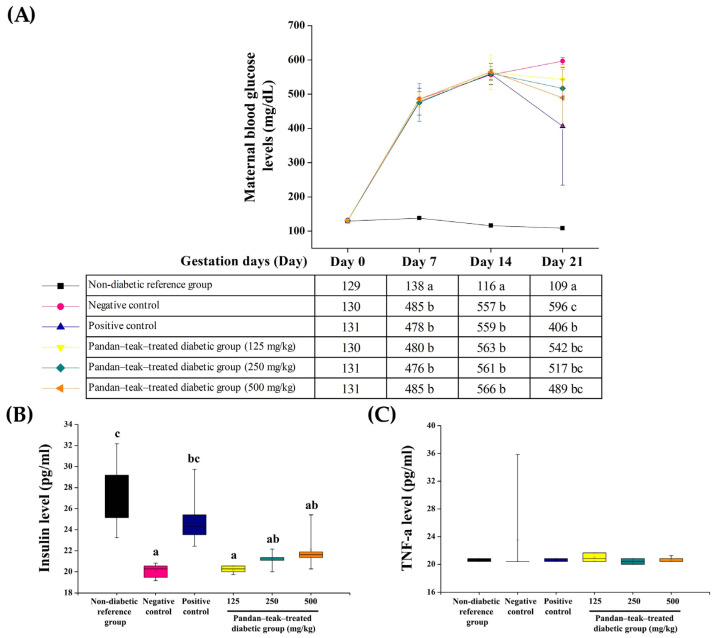
Effects of the pandan–teak formulation on maternal glycemia, insulin, and TNF-α in STZ-induced gestational diabetes. (**A**) Fasting blood glucose levels measured on gestational days (GD) 0, 7, 14, and 21. The untreated diabetic group (negative control) maintained significantly higher glucose levels than the non-diabetic reference group (*p* < 0.05). (**B**) Serum insulin and (**C**) TNF-α concentrations measured on GD 21. Neither the metformin-treated group (positive control) nor the pandan–teak–treated diabetic groups (125, 250, or 500 mg/kg) significantly altered maternal glucose, insulin, or TNF-α levels compared with the untreated diabetic group. Values are expressed as mean ± SD (*n* = 5 refers to the number of dams). Different letters indicate significant differences among groups as determined by one-way ANOVA followed by Tukey’s post hoc test (*p* < 0.05).

Fasting blood glucose levels in the untreated diabetic group (negative control) remained significantly elevated throughout gestation compared with the non-diabetic reference group (*p* = 0.000). Treatment with the pandan–teak–treated diabetic groups did not significantly reduce maternal glucose at any dose, whereas the metformin-treated group (positive control) produced a significant reduction on gestation day 21 (406.40 ± 172.15 mg/dL, *p* = 0.012). Serum insulin and TNF-α concentrations showed no significant differences among treated diabetic groups, indicating that fetal improvements were not mediated through maternal glycemic correction or systemic anti-inflammatory effects. The results are presented in Figure 2.

### 2.3. Maternal Body Weight and Pancreatic Mass Index

**Figure 3:**
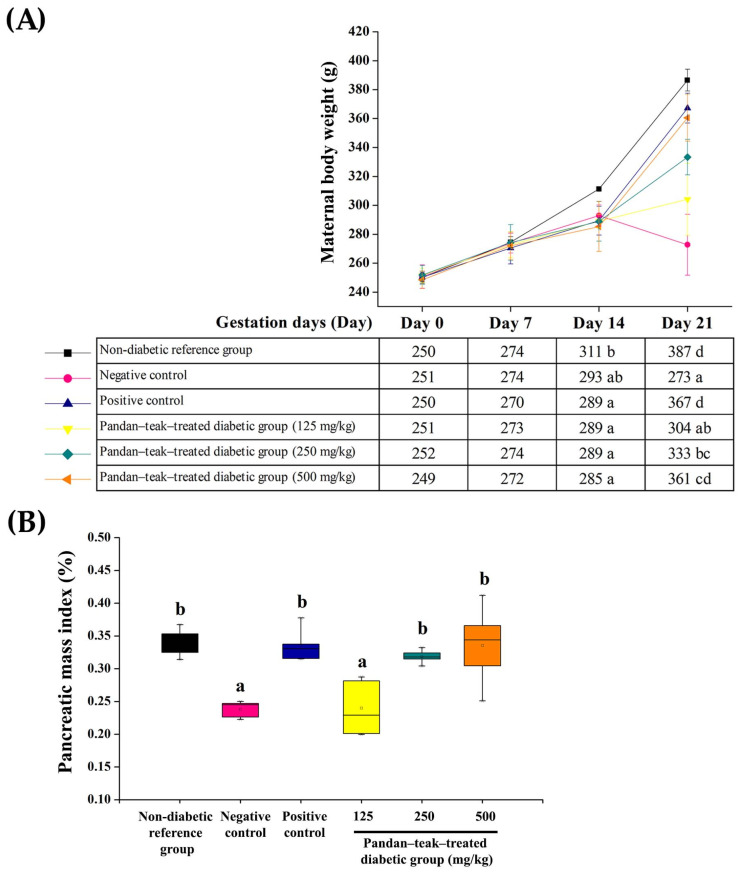
Effects of the pandan–teak formulation on maternal body weight and pancreatic mass index in STZ-induced gestational diabetes. (**A**) Maternal body weight gain and (**B**) pancreatic mass index (pancreas weight/body weight × 100). The untreated diabetic group (negative control) exhibited significant reductions in both parameters compared with the non-diabetic reference group (*p* < 0.05). Treatment with the pandan–teak formulation at medium (250 mg/kg) and high (500 mg/kg) doses significantly improved maternal body weight gain and pancreatic mass index, with effects comparable to the metformin-treated group (positive control), whereas the low-dose group showed no significant change. Data are expressed as mean ± SD (*n* = 5 refers to the number of dams). Bars with different letters indicate significant differences among groups (*p* < 0.05).

Maternal body weight increased progressively in all groups during pregnancy. The untreated diabetic group (negative control) exhibited significantly reduced weight gain compared with the non-diabetic reference group on gestation day 21 (272.74 ± 21.03 g and 386.51 ± 7.48 g, respectively, *p* = 0.000). Administration of the pandan–teak–treated diabetic groups at medium (250 mg/kg) and high (500 mg/kg) doses significantly improved maternal body weight (333.28 ± 12.26 g and 360.53 ± 16.22 g, respectively, *p* = 0.000) and pancreatic mass index (0.32 ± 0.01%, *p* = 0.001 and 0.33 ± 0.06%, respectively, *p* = 0.000), producing effects comparable to those of the metformin-treated group (positive control). The low-dose group showed no significant differences relative to the untreated diabetic group. These results suggest that the formulation preserved maternal metabolic stability and pancreatic integrity without altering glycemic levels. The results are presented in Figure 3.

### 2.4. Fetal Growth Parameters

**Figure 4:**
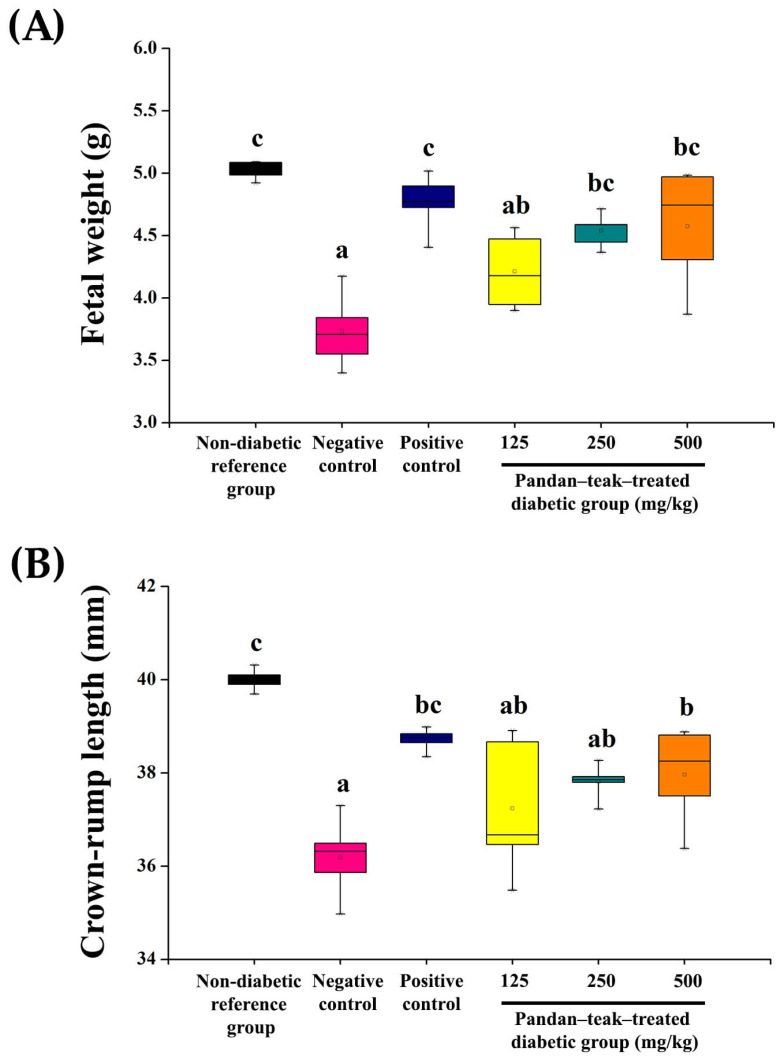
Fetal growth parameters following treatment with the pandan–teak formulation in STZ-induced gestational diabetes. (**A**) Fetal weight (g) and (**B**) crown–rump length (mm) were significantly reduced in the untreated diabetic group (negative control) compared with the non-diabetic reference group (*p* < 0.05). Treatment with the pandan–teak formulation at medium (250 mg/kg) and high (500 mg/kg) doses restored fetal weight in a dose-dependent manner, whereas only the high dose significantly increased crown–rump length. The metformin-treated group (positive control) produced comparable improvements. Values are expressed as mean ± SD (*n* = 5 refers to the number of dams). Fetal measurements were averaged per litter. Bars with different letters indicate significant differences among groups (*p* < 0.05).

Diabetes markedly reduced fetal weight (3.73 ± 0.29 g) and crown–rump length (36.19 ± 0.85 mm) in the untreated diabetic group (negative control) compared with the non-diabetic reference group (5.03 ± 0.07 g, 39.98 ± 0.24 mm, respectively, *p* = 0.000). Treatment with the pandan–teak–treated diabetic groups at medium and high doses significantly restored fetal weight in a dose-dependent manner (4.54 ± 0.14 g, *p* = 0.002 and 4.57 ± 0.48 g, *p* = 0.001, respectively), while only the high dose significantly increased crown–rump length compared with the untreated diabetic group (37.97 ± 1.04 mm, *p* = 0.030). The metformin-treated group (positive control) produced comparable improvements in both fetal weight and crown–rump length (4.77 ± 0.23 g, *p* = 0.000 and 38.72 ± 0.24 mm, *p* = 0.001, respectively). The low-dose formulation did not significantly influence these parameters. These findings demonstrate that the combined extract enhances fetal growth outcomes despite persistent maternal hyperglycemia. The results are presented in Figure 4.

### 2.5. Placental Weight and Placental Index

**Figure 5:**
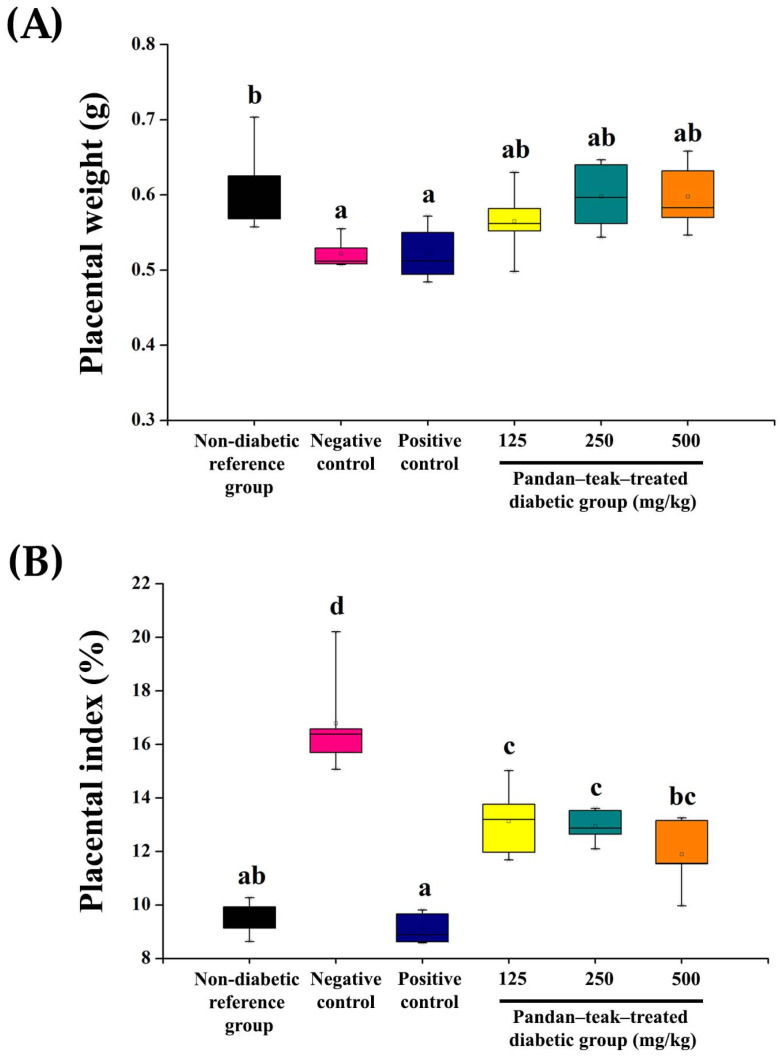
Placental outcomes and fetoplacental efficiency. (**A**) Placental weight (g) and (**B**) placental index (placental weight/fetal weight × 100). All diabetic groups showed lighter placentas than non-diabetic reference group (*p* < 0.05). Although placental weight showed a non-significant upward trend after treatment, placental index was markedly elevated in the untreated diabetic group (negative control), reflecting decreased fetoplacental efficiency. Both metformin-treated group (positive control) and the pandan–teak formulation significantly reduced placental index in a dose-dependent manner (*p* < 0.05), with the high-dose group reaching values comparable to the non-diabetic reference group. Values are expressed as mean ± SD (*n* = 5 refers to the number of dams). Placental data were averaged per litter. Bars with different letters differ significantly (*p* < 0.05).

All diabetic groups exhibited lighter placentas (0.52 ± 0.02 g) than the non-diabetic reference group (0.61 ± 0.06 g, *p* = 0.038). Although the metformin-treated group (positive control) and the pandan–teak-treated diabetic groups increased placental weight modestly, the differences were not statistically significant. In contrast, placental index (placental weight/fetal weight × 100) was markedly elevated in the untreated diabetic group (negative control) (16.79 ± 2.00%, *p* = 0.000), indicating reduced fetoplacental efficiency. Treatment with the metformin-treated group and all doses of the pandan–teak formulation significantly decreased the placental index in a dose-dependent manner (9.12 ± 0.58%, *p* = 0.000, 13.13 ± 1.36%, *p* = 0.001, 12.95 ± 0.63%, *p* = 0.001, and 11.90 ± 1.36%, *p* = 0.000, respectively), with the high-dose group reaching values comparable to those of the non-diabetic reference group (9.54 ± 0.65%). The normalization of placental index suggests improved nutrient transfer efficiency and reduced placental hypertrophy under diabetic conditions. The results are presented in Figure 5.

### 2.6. Fetal Blood Glucose

**Figure 6:**
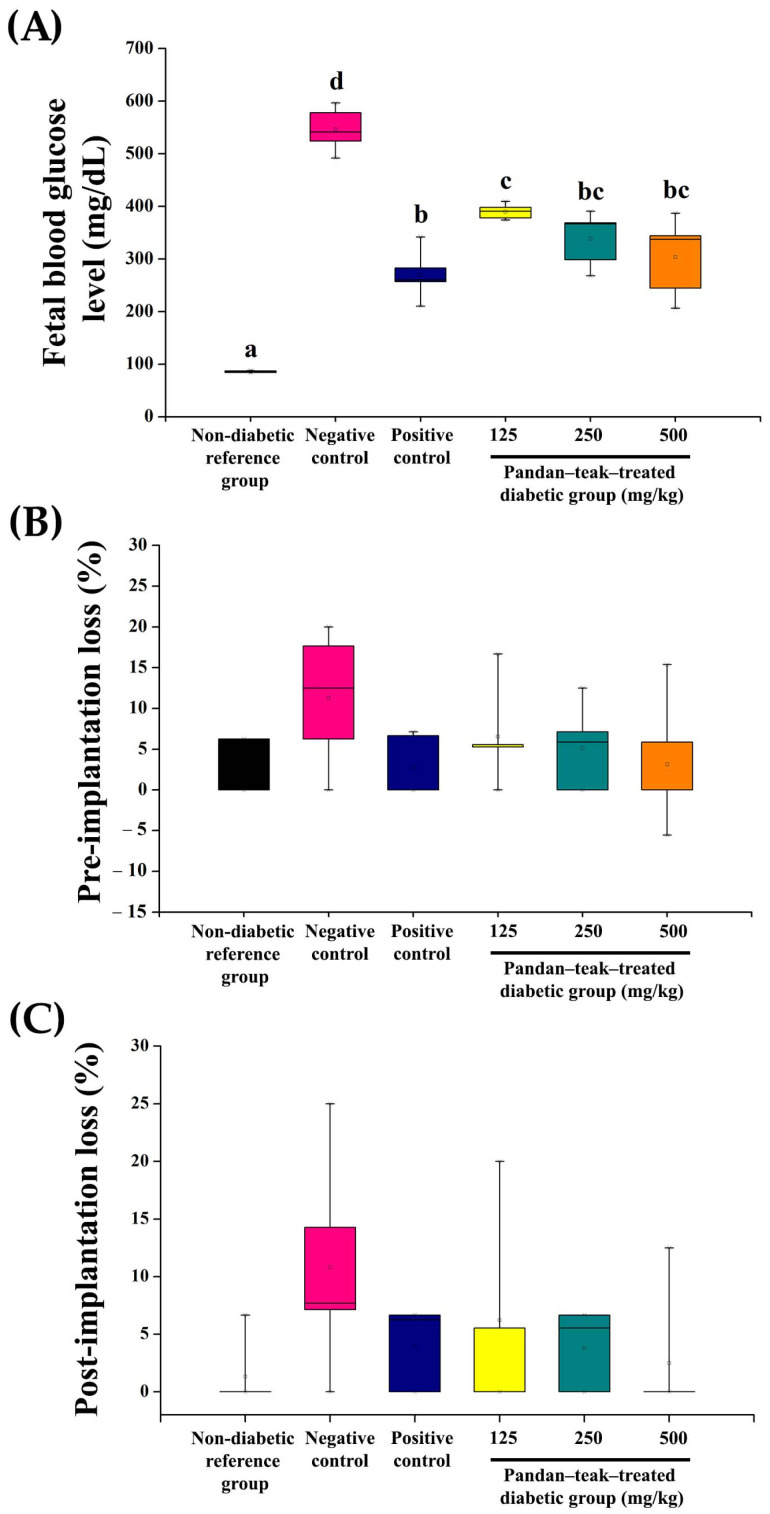
Fetal blood glucose and implantation outcomes. (**A**) Fetal blood glucose concentrations measured on GD 21. Untreated diabetic group (negative control) significantly increased fetal glucose (*p* < 0.05); metformin-treated group (positive control) or the pandan–teak formulation reduced glucose levels to near-normal values in a dose-dependent manner. (**B**,**C**) Pre- and post-implantation loss rates expressed as percentages. Both parameters were elevated in the untreated diabetic group (negative control) but improved following treatment. Data are mean ± SD (*n* = 5 refers to the number of dams). Bars with different letters differ significantly (*p* < 0.05).

Fetal blood glucose levels were markedly elevated in the untreated diabetic group (negative control) (546.30 ± 41.88 mg/dL, *p* = 0.000). Treatment with the metformin-treated group (positive control) and all doses of the pandan–teak–treated diabetic groups significantly reduced fetal glucose levels relative to the untreated diabetic group (270.52 ± 47.79 mg/dL, 390.14 ± 14.42 mg/dL, 338.69 ± 52.36 mg/dL, 303.98 ± 75.24 mg/dL, respectively, *p* = 0.000). The effect was dose-dependent, and the high-dose group achieved glucose levels statistically comparable to those of the non-diabetic reference group (85.87 ± 1.62 mg/dL). This reduction in fetal glycemia, despite unchanged maternal glucose, indicates selective modulation of placental glucose transport or fetal glucose utilization. The results are presented in Figure 6.

### 2.7. Implantation Loss and Fetal Viability

Pre-implantation and post-implantation loss rates were higher in the untreated diabetic group (negative control) (11.28 ± 8.23% and 10.82 ± 9.40%, respectively) than in the non-diabetic reference group (2.50 ± 3.42%, *p* = 0.240 and 1.33 ± 2.98%, *p* = 0.171, respectively). Treatment with the pandan–teak–treated diabetic groups and the metformin-treated group (positive control) improved implantation success and fetal viability, although differences among treated groups did not reach statistical significance. No treatment-related malformations or external anomalies were observed in fetuses from any group, suggesting that the polyherbal formulation did not adversely affect embryonic development. The results are presented in Figure 6.

### 2.8. Summary of Key Findings

The pandan–teak formulation improved multiple fetal outcomes in diabetic pregnancy without altering maternal glycemia. Medium and high doses enhanced fetal growth, reduced placental index, and normalized fetal blood glucose. These results point to a unique placental–fetal regulatory mechanism, potentially involving antioxidant and metabolic synergism between pandan and teak phytochemicals.

## 3. Discussion

This study provides novel evidence that a polyherbal formulation combining *Pandanus amaryllifolius* root and *Tectona grandis* leaf extracts improves fetal outcomes in streptozotocin (STZ)-induced gestational diabetes mellitus (GDM) without altering maternal glycemia. The key findings include dose-dependent increases in fetal weight and crown–rump length, normalization of fetal glucose levels, and restoration of placental efficiency, with medium and high doses producing effects comparable to metformin-treated group. These results suggest that the pandan–teak formulation may exert protective effects at the placental–fetal interface rather than through direct maternal glucose lowering.

### 3.1. Maternal Metabolic Response

In normal pregnancy, physiological insulin resistance increases to ensure adequate glucose availability for fetal growth [18]. In GDM, β-cell compensation is insufficient, resulting in sustained maternal hyperglycemia and oxidative stress [19,20]. Consistent with previous STZ models, untreated diabetic dams in this study exhibited reduced body weight and pancreatic mass, reflecting β-cell cytotoxicity. Administration of medium and high doses of the pandan–teak formulation significantly improved maternal weight gain and pancreatic mass index, suggesting partial preservation of β-cell function and energy balance. However, maternal glycemia and insulin remained unchanged, indicating that the formulation’s primary effects were not mediated through systemic glucose correction but likely through improved oxidative status and nutrient utilization efficiency [21].

### 3.2. Fetal Outcomes and Placental Efficiency

Maternal diabetes adversely affects placental structure and function, leading to fetal overgrowth or growth restriction depending on the balance between hyperglycemia and vascular insufficiency [22]. In this study, fetuses of diabetic rats exhibited reduced weight and crown–rump length, consistent with placental insufficiency. The pandan–teak formulation improved both parameters in a dose-dependent manner, paralleling a significant reduction in placental index, which reflects enhanced fetoplacental efficiency [23]. The normalization of fetal glucose levels despite persistent maternal hyperglycemia suggests selective regulation of placental glucose transport or improved fetal glucose utilization. These results align with previous findings that antioxidant and anti-inflammatory interventions can restore placental morphology and support fetal growth under diabetic stress [24,25,26].

### 3.3. Mechanistic Considerations at the Placental–Fetal Interface

The present findings demonstrate that the pandan–teak formulation significantly improves fetal growth parameters, reduces placental index, and normalizes fetal glucose levels in STZ-induced gestational diabetes, despite the absence of significant changes in maternal glycemia. This dissociation between maternal and fetal glucose regulation suggests that the formulation may exert its primary effects at the placental–fetal interface rather than through direct maternal glucose lowering.

Although the precise molecular mechanisms were not directly examined in this study, the observed fetal-protective effects are consistent with previously reported biological activities of *Pandanus amaryllifolius* and *Tectona grandis*. Pandan extracts have been reported to exhibit insulinotropic, α-glucosidase-inhibitory, and glucose-uptake-enhancing properties, while teak leaf extracts are rich in polyphenolic compounds with established antioxidant and anti-inflammatory activities. It is therefore plausible that the combined formulation may support fetal growth by modulating placental oxidative stress, inflammatory signaling, or glucose handling, thereby improving fetoplacental efficiency under diabetic conditions.

Importantly, the normalization of fetal glucose levels in the absence of maternal glycemic correction suggests a selective regulatory effect on placental glucose transfer or fetal glucose utilization. Previous studies have shown that placental oxidative stress and inflammatory mediators can disrupt glucose transporter function and nutrient exchange in diabetic pregnancy. Interventions that reduce oxidative or inflammatory stress at the maternal–fetal interface have been reported to restore placental efficiency and improve fetal metabolic outcomes without necessarily normalizing maternal blood glucose. The present findings are consistent with this paradigm.

Nevertheless, it must be emphasized that the antioxidant, anti-inflammatory, and insulinotropic actions proposed here remain hypothetical and are inferred from physiological outcomes and existing literature rather than direct mechanistic measurements. The current study did not assess oxidative stress biomarkers, inflammatory cytokines beyond systemic TNF-α, insulin signaling pathways, or placental glucose transporter expression. Future studies incorporating molecular and histological analyses of placental and fetal tissues will be essential to confirm the mechanistic basis underlying the observed fetal benefits.

### 3.4. Comparison with Metformin

Metformin, a first-line antidiabetic drug, acts primarily through suppression of hepatic gluconeogenesis and enhancement of insulin sensitivity. In GDM models, it reduces maternal glucose and modulates placental nutrient transporters. Interestingly, both metformin and the pandan–teak formulation normalized fetal glucose levels.

It should also be noted that metformin is unlikely to markedly reduce maternal glycemia in STZ models characterized by extensive β-cell destruction, which may explain why maternal glucose levels remained elevated across all diabetic groups despite treatment.

It should be emphasized that persistent maternal hyperglycemia is clinically undesirable and is associated with increased maternal risks. Therefore, the present findings do not suggest that the pandan–teak formulation could replace standard glycemic control. Rather, it may be considered a potential adjunct therapy aimed at improving placental–fetal outcomes alongside established treatments.

Metformin is widely used in the clinical management of gestational diabetes and has been shown to improve maternal and neonatal outcomes; however, it readily crosses the placenta, and its long-term metabolic effects on offspring remain under active investigation. While the present formulation produced comparable improvements in fetal glucose levels and placental index, the safety of herbal formulations during pregnancy likewise requires rigorous toxicological and clinical evaluation before clinical application.

Unlike metformin, which crosses the placenta via organic cation transporters and has uncertain long-term safety in offspring, the pandan–teak formulation—composed of edible phytochemicals—may provide a safer alternative for managing diabetic pregnancy, pending toxicological confirmation [27].

### 3.5. Clinical and Translational Implications

These findings provide experimental support for the potential of *Pandanus amaryllifolius* and *Tectona grandis*–based formulations to improve fetal outcomes under diabetic conditions. The observed improvements in fetal growth and fetal glycemia, in the absence of maternal glucose normalization, suggest a possible role as a supportive adjunct approach targeting placental–fetal function rather than primary glycemic control. However, translation of these findings to clinical practice requires caution, as safety, dosing, and efficacy in human pregnancy remain to be established. The polyherbal concept may offer theoretical advantages through the combined actions of multiple bioactive constituents, but further mechanistic, toxicological, and clinical studies are necessary before any therapeutic recommendations can be made [28].

### 3.6. Limitations and Future Directions

A key limitation of this study is the use of a single high-dose STZ injection on gestational day 5 to induce gestational diabetes. This model produces substantial pancreatic β-cell loss and therefore represents an extreme form of GDM with β-cell failure, which more closely resembles severe diabetes than the typical insulin-resistant phenotype of human gestational diabetes. Consequently, caution is required when extrapolating these findings directly to clinical GDM.

In addition, the present study did not include treatment groups receiving *Pandanus amaryllifolius* or *Tectona grandis* extracts individually. As a result, the relative contributions of each botanical cannot be distinguished, and potential synergistic actions between pandan and teak were not formally demonstrated. The observed effects should therefore be interpreted as arising from the collective actions of constituents in the combined formulation.

From a methodological perspective, statistical analyses were performed using one-way ANOVA with the dam treated as the experimental unit. Although repeated-measures or mixed-effects models would be more appropriate for longitudinal maternal outcomes and proportional implantation data, the limited sample size constrained the application of more complex statistical approaches. This should be considered when interpreting the results.

Relatedly, the sample size of five dams per group was relatively small. While large effect sizes allowed several outcomes to reach statistical significance, some variables, including TNF-α levels and implantation loss, may have been underpowered. Accordingly, nonsignificant findings should be interpreted with caution.

A further limitation of the present study is the absence of a comprehensive systemic metabolic characterization of maternal and fetal biochemical status. Key parameters reflecting longer-term glycemic control and broader metabolic alterations, such as fructosamine and basic plasma biochemistry, were not assessed. This reflects the focused scope of the current work, which prioritized fetal growth, fetal glycemia, and placental efficiency rather than extensive metabolic profiling. Future studies should incorporate these biochemical markers to provide a more complete understanding of maternal and fetal metabolic adaptations in gestational diabetes.

Finally, the current study was limited by its relatively short gestational treatment window and the lack of molecular or histological analyses confirming oxidative stress modulation, inflammatory signaling, or placental glucose transporter activity. Future investigations should include quantitative assessments of oxidative and inflammatory biomarkers, evaluation of placental glucose transporters (e.g., GLUT1 and GLUT3), and long-term postnatal follow-up of offspring to assess metabolic programming effects. Ultimately, well-designed human studies will be essential to confirm safety, optimal dosing, and translational relevance.

## 4. Materials and Methods

### 4.1. Plant Materials and Extraction

Roots of *Pandanus amaryllifolius* Roxb. and leaves of *Tectona grandis* L.f. were collected from authenticated sources previously described in our earlier work [29]. Extraction followed optimized procedures for each plant: pandan roots were extracted with hot water (70 °C, 8 h; yield = 11.8%), while teak leaves were extracted with 70% ethanol under reflux (45 °C; yield = 9.4%). Filtrates were concentrated under reduced pressure at 45 °C and stored at 4 °C until use.

### 4.2. Phytochemical Analysis of Extracts

Phytochemical profiles of the individual extracts were characterized previously using gas chromatography–mass spectrometry (GC–MS). Retention times, peak areas, and compound identifications were validated as reported in our prior publication [29]. Compounds were tentatively identified by comparing their mass spectra with those in the NIST mass spectral library and by matching retention times with published data. Relative abundances were calculated based on peak area normalization. Compound identification was therefore putative and based on spectral similarity rather than confirmation with authentic standards. Detailed GC–MS profiles of the extracts are provided in Supplementary Materials, Table S1.

### 4.3. Preparation of the Polyherbal Formulation

A fixed 1:1 (*w*/*w*) ratio of pandan root and teak leaf extracts was used to prepare the formulation. Extracts were dissolved in purified water to achieve final concentrations of 125, 250, and 500 mg/kg. The administered volume varied slightly with solubility but was standardized to deliver consistent total doses across all groups.

### 4.4. Animal Ethics Statement

All animal procedures were approved by the Institutional Animal Care and Use Committee (IACUC) of Suranaree University of Technology (Protocol No. SUT-IAUCUC-008/2022) and complied with national and institutional ethical standards. Humane endpoints were predefined, and all euthanasia procedures minimized distress.

### 4.5. Experimental Animals and Induction of Gestational Diabetes

Ninety female Wistar rats (3–6 months old, 250–300 g) were housed under controlled environmental conditions (12 h light/dark, 20 ± 5 °C, 45 ± 5% humidity) with ad libitum access to food and water. After one week of acclimatization, females were paired with fertile males (2:1) overnight; sperm-positive smears confirmed mating (gestational day 0, GD 0).

On GD 5, gestational diabetes was induced by intraperitoneal injection of streptozotocin (STZ; 60 mg/kg in 0.1 M citrate buffer, pH 6.5) prepared immediately before use. Blood glucose was measured 48 h post-injection using a calibrated glucometer (Accu-Chek Performa, Roche Diagnostics, Bangkok, Thailand). Rats with fasting glucose > 200 mg/dL were classified as hyperglycemic and included in treatment protocols, consistent with validated GDM induction models [8,30].

Following confirmation of gestational diabetes, animals were randomly allocated to experimental groups using a simple randomization procedure. Outcome assessments were performed by investigators blinded to group assignments.

### 4.6. Treatment Protocol

Following model confirmation, diabetic rats were randomly assigned to experimental groups consisting of an untreated diabetic group (negative control), a metformin-treated diabetic group (positive control; 300 mg/kg), and diabetic groups receiving the pandan–teak formulation at doses of 125, 250, or 500 mg/kg body weight. Non-diabetic pregnant rats served as a non-diabetic reference group (*n* = 5 dams). The diabetic group was further divided into four treatment subgroups, each comprising 10–15 rats. Doses were selected based on previous toxicity and efficacy data [27,31,32]. All treatments were administered orally by gavage once daily from GD 7 to GD 21 using flexible feeding needles to prevent injury.

Maternal body weight and food intake were recorded daily. Food consumption was calculated as the difference between offered and remaining feed after 24 h. Dams were also monitored for vaginal bleeding or discharge indicative of gestational complications.

### 4.7. Animal Welfare and Monitoring

All manipulations adhered to international welfare guidelines. Trained personnel conducted twice-daily health checks for behavioral and physiological signs of distress. Expected transient symptoms (polyuria, polydipsia, mild weight loss) were documented. Humane endpoints included ≥15% body-weight loss, persistent anorexia or dehydration, or unrelieved pain. Euthanasia was performed in accordance with IACUC standards following deep anesthesia, using carbon dioxide overdose and cervical dislocation where appropriate.

### 4.8. Blood Glucose Measurement

Fasting blood glucose was measured after an overnight fasting period of 10–12 h on gestational days (GD) 0, 7, 14, and 21. Glucose measurements were obtained in the morning (08:00–10:00 h) by tail-vein puncture using a handheld glucometer. Calibration was verified with manufacturer control solutions to ensure reliability of longitudinal glucose data.

### 4.9. Serum Insulin and TNF-α Determination

On GD 21, dams were deeply anesthetized, and blood samples were collected by terminal cardiac puncture. Euthanasia was completed immediately thereafter in accordance with institutional ethical guidelines. Serum was separated by centrifugation (2500 rpm, 12 min, 4 °C) and stored at −80 °C. Insulin and TNF-α levels were quantified by ELISA using Rat Insulin (FineTest^®^, Wuhan Fine Biotech Co., Ltd., Wuhan, China) and Rat TNF-α (Abcam, Abcam Singapore Pte. Ltd., The Metropolis Tower Two, Singapore) kits. Absorbance was read at 450 nm with a Multiskan™ GO microplate reader (Thermo Fisher Scientific Inc., Waltham, MA, USA), and concentrations were calculated using cubic-spline regression. According to the manufacturers’ specifications, the intra-assay and inter-assay coefficients of variation for the insulin and TNF-α ELISA kits were <10% and <10%, respectively.

### 4.10. Maternal and Fetal Outcome Measurements

On GD 21, a midline laparotomy was performed to excise the gravid uterus, which was weighed to assess total reproductive burden. Both ovaries were examined to count the number of corpora lutea (CL), while each uterine horn was examined for implantation sites, resorptions, and live or dead fetuses. *Pre-implantation loss rate* was calculated using the equation (*CL* − *implantations*)/*CL* × 100, where *CL* denotes the number of corpora lutea and *implantations* represents the number of implantation sites. *Post-implantation loss rate* was calculated as (*implantations* − *live fetuses*)/*implantations* × 100, where *live fetuses* indicates the number of viable fetuses.

Each fetus and placenta were weighed individually. To account for variation in litter size, fetal and placental measurements were summarized per litter, with each dam treated as the experimental unit. Mean fetal weight, crown–rump length, and fetal glucose levels were calculated for each litter prior to statistical analysis, and individual fetuses were not pooled across dams.

Placental index (%) = (placental weight/fetal weight) × 100, representing placental efficiency under altered maternal metabolic conditions [33]. Fetal blood glucose was measured immediately postmortem from jugular samples using the same glucometer protocol as for maternal assessment.

### 4.11. Statistical Analysis

All quantitative data are expressed as mean ± SD. Categorical outcomes (e.g., viability, resorption rate) are presented as percentages. Group means were compared using one-way ANOVA followed by Tukey’s post hoc test when *p* < 0.05. Statistical analyses were performed in SPSS 17.0 (SPSS Inc., Chicago, IL, USA), and graphical plots were generated using Origin 8.5 (OriginLab, Northampton, MA, USA). For all analyses, the dam was considered the experimental unit. Fetal and placental measurements were averaged per litter prior to statistical analysis. Fetal and placental measurements were summarized per litter, with each dam treated as the experimental unit for statistical analysis.

## 5. Conclusions

This study demonstrates that a polyherbal formulation combining *Pandanus amaryllifolius* root and *Tectona grandis* leaf extracts improves fetal growth and fetal glucose homeostasis in a streptozotocin-induced model of gestational diabetes, without significantly altering maternal glycemia. Medium and high doses increased fetal weight and crown–rump length, reduced the placental index, and normalized fetal glucose levels, indicating improved placental–fetal efficiency. These benefits may reflect the collective actions of antioxidant, anti-inflammatory, and insulinotropic constituents present in the formulation, which may contribute to the protection of β-cell and placental function.

Importantly, these findings provide experimental support for the traditional use of pandan and teak in promoting maternal metabolic balance and fetal development. The formulation’s ability to improve fetal outcomes despite persistent maternal hyperglycemia suggests its potential as a plant-based adjunct approach aimed at supporting placental–fetal health alongside established glycemic management strategies, rather than as a replacement for standard pharmacological therapy.

Future research should further elucidate the underlying molecular mechanisms, particularly those related to oxidative stress modulation, placental glucose transporter regulation, and mitochondrial metabolism. In addition, postnatal and transgenerational outcomes in offspring, as well as comprehensive toxicological and clinical evaluations, will be essential to confirm safety, efficacy, and translational relevance of this formulation as a fetal-protective strategy in diabetic pregnancy.

## Data Availability

The datasets generated and/or analyzed during the current study are available in the Mendeley Data repository under the title “Raw data for *Pandanus amaryllifolius* and *Tectona grandis* study” [https://doi.org/10.17632/2w7rbpb4v9.1]. The repository includes raw numerical data underlying all figures and tables output files.

## Supplementary Materials

The following supporting information can be downloaded at: https://www.mdpi.com/article/10.3390/ijms27020857/s1.

## Abbreviations

The following abbreviations are used in this manuscript:

GDM	Gestational diabetes mellitus
STZ	Streptozotocin
TNF-α	Tumor necrosis factor-α
HOMA-IR	Homeostatic model assessment of insulin resistance
GD	Gestational days
SD	Standard deviation
GLUT	Glucose transporters
GC-MS	Gas chromatography–mass spectrometry
w/w	Weight per weight
IACUC	Institutional Animal Care and Use Committee
SUT	Suranaree University of Technology
CL	Corpora lutea
ANOVA	One-way analysis of variance
