# Supplementary material for: Pandanus amaryllifolius and Tectona grandis Extracts Improve Fetal Outcomes in Streptozotocin-Induced Gestational Diabetes in Rats
Source: Int J Mol Sci. 2026 Jan 15;27(2):857. doi: 10.3390/ijms27020857 (PMC12840670; doi:10.3390/ijms27020857)
Supplement: Supplementary file 1 [file ijms-27-00857-s001.zip › ijms-4020311-supplementary.pdf]

Supplementary Material:

Table S1. Gas Chromatography-Mass Spectrometry analysis report of pandan and teak extracts with the relevant details.

No.	Peak name	Retention Time (min)	Peak area (%)
1	Propanoic acid, 3,3'-thiobis-, didodecyl ester	53.013	8.37
2	Lupeol	45.647	0.13
3	β-Sitosterol	44.178	1.90
4	Stigmasterol	43.142	1.86
5	Campesterol	42.695	0.48
6	Dehydrodiconiferyl alcohol	42.302	0.15
7	Isosesamin	41.769	0.04
8	4,8,13-Duvatriene-1,3-Diol	39.198	0.14
9	Squalene	37.533	0.06
10	α-Monostearin	36.244	0.07
11	Heptacosane	36.067	0.18
12	Pregnenolone	35.991	0.07
13	Butyl 9,12,15-octadecatrienoate	35.892	0.28
14	β-Monolinolein	35.786	0.07
15	10,11-Dihydro-10-hydroxy-2,3-dimethoxydibenz(b,f)oxepin	35.133	0.16
16	Diisooctyl phthalate	33.583	0.54
17	Glycerol β-palmitate	33.327	0.26
18	Enantio-Polyalthic acid	32.580	0.82
19	Anticopalic acid	31.539	6.18
20	Eicosanoic acid	31.227	0.07
21	Methyl copalate	31.010	7.07
22	Catavic acid	30.194	10.61
23	Epi-13-Manool	29.306	0.21
24	Octadecanoic acid	28.374	1.39
25	Linolenic acid	28.058	2.47

26	Linoleic acid	27.919	1.10
27	Phytol	27.599	0.87
28	Methyl 1,4-dihydroxy-2-naphthoate	27.057	0.07
29	Heptadecanoic acid	26.951	0.14
30	Propanoic acid, 3-mercapto-, dodecyl ester	26.341	1.54
31	n-Hexadecanoic acid	25.698	3.67
32	Lidocaine	24.700	0.32
33	3,5-Dimethoxy-4-hydroxyphenethylamine	24.663	0.08
34	Platambin	24.424	0.16
35	Pentadecanoic acid	24.358	0.09
36	Acetosyringone	23.779	0.08
37	β-Hydroxypropiovanillone	23.740	0.12
38	Tetradecanoic acid	23.061	0.32
39	Tryptophol	22.992	0.07
40	4-((1E)-3-Hydroxy-1-propenyl)-2-methoxyphenol	22.751	1.46
41	Dodecyl acrylate	22.140	5.00
42	Homovanillic acid	21.443	0.24
43	1-Dodecanol	18.921	2.32
44	Benzene, 1-(bromomethyl)-3-nitro-	18.407	0.83
45	Vanillin	17.638	0.07
46	Phenol, 2,6-dimethoxy-	16.816	0.34
47	2-Methoxy-4-vinylphenol	16.191	0.49
48	Indole	15.875	0.28
49	1,2-Ethanediol, 1-(2-furanyl)-	14.983	0.69
50	5-Hydroxymethylfurfural	14.787	0.29
51	Benzofuran, 2,3-dihydro-	14.580	0.28
52	Catechol	14.274	0.86
53	5-Hydroxymaltol	14.085	0.09
54	Benzoic acid	13.880	0.84
55	2,4-Hexadienedioic acid	13.567	0.34

56	Pyranone	13.234	1.27
57	Phenylethyl Alcohol	12.491	0.55
58	Maltol	12.427	0.13
59	Phenol, 2-methoxy-	11.924	0.82
60	2,5-Dimethyl-4-hydroxy-3(2H)-furanone	11.679	0.17
61	2(3H)-Furanone, 5-ethoxydihydro-	11.469	0.04
62	1-Amino-2,6-dimethylpiperidine	11.114	0.06
63	Benzyl alcohol	10.928	0.10
64	Phenol	9.845	0.69
65	2,4-Dihydroxy-2,5-dimethyl-3(2H)-furan-3-one	9.770	0.21
66	2(5H)-Furanone, 3-methyl-	9.611	0.34
67	5-Methyl furfural	9.362	0.12
68	2(3H)-Furanone, dihydro-3-methyl-	9.088	0.24
69	1,2-Cyclopentanedione	8.660	0.41
70	Butanoic acid, 4-hydroxy-	8.294	0.17
71	4-Cyclopentene-1,3-dione	7.659	0.14
72	2-Propanone, 1-(acetyloxy)-	7.462	0.14
73	2-Furanmethanol	7.174	0.42
74	Propanoic acid, 2,3-dihydroxy-	6.923	1.09
75	Furfural	6.721	0.29
76	Glycolaldehyde dimethyl acetal	6.248	0.70
77	2,3-Butanediol	6.224	19.02
78	Propanoic acid, 2-oxo-, methyl ester	5.969	0.23
79	2-Propenoic acid, 2-hydroxyethyl ester	5.286	0.65
80	2-Propenoic acid	4.745	0.21
81	2-Propanone, 1-hydroxy-	4.449	0.44
82	Acetic acid	4.204	4.75
